# Plasma Exosome Proteins ILK1 and CD14 Correlated with Organ-Specific Metastasis in Advanced Gastric Cancer Patients

**DOI:** 10.3390/cancers15153986

**Published:** 2023-08-05

**Authors:** Chenfei Zhou, Changting Qiao, Jun Ji, Wenqi Xi, Jinling Jiang, Liting Guo, Junwei Wu, Feng Qi, Qu Cai, Steven W. M. Olde Damink, Jun Zhang

**Affiliations:** 1 Department of Oncology, Ruijin Hospital, Shanghai Jiao Tong University School of Medicine, Shanghai 200025, China; zcf12085@rjh.com.cn (C.Z.); xwq11061@rjh.com.cn (W.X.); jjl11872@rjh.com.cn (J.J.); glt12250@rjh.com.cn (L.G.); wjw11700@rjh.com.cn (J.W.); qf12486@rjh.com.cn (F.Q.); cq40546@rjh.com.cn (Q.C.); 2 Department of Oncology, Wuxi Branch of Ruijin Hospital, Shanghai Jiao Tong University School of Medicine, Wuxi 214111, China; 3 Department of Surgery, NUTRIM School of Nutrition and Translational Research in Metabolism, Maastricht University, 6200 MD Maastricht, The Netherlands; steven.oldedamink@maastrichtuniversity.nl; 4 Department of Pathology, Ruijin Hospital, Shanghai Jiao Tong University School of Medicine, Shanghai 200025, China; qct40479@rjh.com.cn; 5 Shanghai Institute of Digestive Surgery, Ruijin Hospital, Shanghai Jiao Tong University School of Medicine, Shanghai 200025, China; jj40464@rjh.com.cn

**Keywords:** gastric cancer, exosome, organ-specific metastasis, tumor microenvironment

## Abstract

**Simple Summary:**

Organ-specific metastasis is a special metastatic mode that can be observed in gastric cancer. The exosome is an important commutator between tumor cells and the microenvironment. Its role in organ-specific metastasis in gastric cancer has not been fully investigated. We aimed to delineate the biological functions of plasma exosome proteins and their correlations during organ-specific metastasis in gastric cancer. The study found that biological functions of plasma exosome proteins among AGC patients with different metastatic modes were distinct, in which ILK1 and CD14 were correlated with organ-specific metastasis and participated in regulating malignant behaviors in gastric cancer cells. Plasma exosome CD14 was derived from immune cells in malignant ascites with high activation of chemokine- and cytokine-mediated signaling pathways.

**Abstract:**

The exosome plays important roles in driving tumor metastasis, while the role of exosome proteins during organ-specific metastasis in gastric cancer has not been fully understood. To address this question, peripheral blood samples from 12 AGC patients with organ-specific metastasis, including distant lymphatic, hepatic and peritoneal metastasis, were collected to purify exosomes and to detect exosome proteins by Nano-HPLC–MS/MS. Gastric cancer cell lines were used for in vitro experiments. Peripheral blood sample and ascites sample from one patient were further analyzed by single-cell RNA sequencing. GO and KEGG enrichment analysis showed different expression proteins of hepatic metastasis were correlated with lipid metabolism. For peritoneal metastasis, actin cytoskeleton regulation and glycolysis/gluconeogenesis could be enriched. ILK1 and CD14 were correlated with hepatic and peritoneal metastasis, respectively. Overexpression of CD14 and ILK1 impacted the colony formation ability of gastric cancer and increased expression of Vimentin. CD14 derived from immune cells in malignant ascites correlated with high activation of chemokine- and cytokine-mediated signaling pathways. In summary, biological functions of plasma exosome proteins among AGC patients with different metastatic modes were distinct, in which ILK1 and CD14 were correlated with organ-specific metastasis.

## 1. Introduction

Gastric cancer is one of the most malignant diseases, with a strong tendency to metastasize to distant organs, including peritoneum, lymph nodes, liver, lung and bone [1]. Tumor metastases can cause multiple complications which significantly impair patients’ outcomes and quality of life. For instance, peritoneal metastasis, which is the most common type of gastric cancer metastasis, is attributed to complications including ascites, bowel obstruction and cachexia and is usually insensitive to chemotherapy [2]. The median overall survival of advanced gastric cancer (AGC) patients with distant metastasis is barely over 12 months in the era of conventional chemotherapy [3]. Therefore, to understand the molecular mechanisms of gastric cancer metastasis, it is critical to diagnose and optimize treatment for those patients.

Development of tumor metastasis is complicated due to its multistep process and multi-factor involvement. The theory of “seeds and soil” provides the modern concepts and explanations of tumor metastasis [4]. However, molecular events driving cancer cells to metastasize to different organs, as well as interactions with distinct microenvironments, increase the difficulty in understanding metastatic mechanisms in patients with multi-organ metastasis. Organ-specific metastasis is a special mode in which tumor cells selectively seed and outgrow in specific organs and can also be observed in AGC patients during clinical practice [5,6]. Focusing on this special metastatic mode may help us to understand the underlying mechanisms of tumor metastasis and to develop strategies for diagnosis and treatment of these lethal conditions.

Interaction between tumor cells and the host microenvironment has been recognized as an important contributing factor in promoting tumor metastasis [7]. In addition to interacting directly, bioactive factors derived from tumor cells (as well as stromal cells) can trigger important signaling pathways of tumor cells and can also help to establish a pre-metastatic niche in distant organs to facilitate tumor metastasis through endocrine and paracrine modes [8]. The exosome is one of the most vital communicators between different cell types to transmit bioactive molecules, which also has been reported to play an important role in driving organ-specific metastasis [9]. With the characteristics of high stability and large presence in body fluid, exosomes can also be potential biomarkers to help with cancer diagnosis [10]. In gastric cancer, multiple investigations have been performed to reveal the roles of exosomes during tumor progression and metastasis [11]. However, the role of proteins packed in plasma exosomes during the process of organ-specific metastasis in gastric cancer has not been fully investigated.

In the present study, we purified plasma exosomes by collecting peripheral blood samples from newly diagnosed AGC patients with organ-specific metastasis and detected exosome proteins by Nano-HPLC–MS/MS. We aimed to delineate the biological functions of plasma exosome proteins and their correlation with organ-specific metastasis in gastric cancer.

## 2. Materials and Methods

### 2.1. Patients and Samples

Peripheral blood samples were collected to purify exosomes from 12 patients who were diagnosed as stomach adenocarcinoma with organ-specific metastasis before the start of systemic treatment in the Department of Oncology, Ruijin Hospital. Patients were stratified into three groups according to their metastatic modes: distant lymph nodes (L group), liver (H group) and peritoneum (P group). Organ-specific metastasis was confirmed by using computer tomography (CT) scans which were reviewed by an experienced oncologist (Figure S1). Plasma samples from an independent cohort of chemo-naïve AGC patients who were diagnosed with organ-specific metastasis were collected for enzyme-linked immunosorbent assay (ELISA) to verify the detected exosome proteins. Peripheral blood samples and ascites samples were collected from one patient with peritoneal metastasis for single-cell RNA sequencing (scRNA-seq) analysis. Informed consent from all subjects was obtained before sample collection. This study was approved by the ethics committee of Ruijin Hospital, Shanghai Jiao Tong University School of Medicine, Shanghai, People’s Republic of China (26 April 2017, No. 61).

### 2.2. Exosome Isolation and Purification

Plasma samples were used for exosome isolation with Umibio^®^ exosome isolation kits (Umibio, Shanghai, China). In brief, an initial spin was performed at 3000× *g*, 4 °C, for 10 min and 10,000× *g*, 4 °C, for 20 min for each sample, to remove cells and debris. The corresponding amounts of reagents were added proportionally to the starting sample volume, according to the manufacturer’s instructions. Mixtures were vortexed and incubated at 4 °C for up to 2 h and then centrifuged at 10,000× *g*, 4 °C, for 60 min to precipitate exosome pellets. Pellets were resuspended with 1 × PBS and purified with Exosome Purification Filter at 3000× *g*, 4 °C, for 10 min. All exosomes were stored at −80 °C immediately after isolation until further analysis. Exosome particle size and concentration were determined using nanoparticle tracking analysis (NTA) at VivaCell Biosceinces with ZetaView PMX 110 (Particle Metrix, Meerbusch, Germany) (Table S1). Western blot was performed to detect exosome biomarkers including CD63 and TSG101 (Abcam, Cambridge, MA, USA). Transmission electron microscopy was performed to visualize exosomes, and the negative staining method was used [12] (Figure S2).

### 2.3. Nano-HPLC–MS/MS Analysis

Samples were lysed by lysis buffer (1% SDS, 8 M urea, 1 × Protease Inhibitor Cocktail (Roche Ltd., Basel, Switzerland)). The protein concentration was determined by BCA assay. Then, 100 μg of protein per condition was transferred into a new Eppendorf tube for protein digestion. The resultant peptide mixture was labeled with TMT-6plex Isobaric Mass Tag Labeling Kit (Thermo Fisher Scientific, Waltham, MA, USA), following the manufacture’s instruction. The peptides were re-dissolved in solvent A (A: 0.1% formic acid in water) and analyzed by on-line nanospray LC–MS/MS on an Orbitrap Fusion™ Tribrid™ mass spectrometer (Thermo Fisher Scientific, Waltham, MA, USA) coupled to a nanoACQUITY UPLC system (Waters Corporation, Milford, MA, USA). Next, 5 μL peptide was loaded (analytical column: Waters nanoEase M/Z HSS C18 T3, 75 μm × 25 cm) and separated with a 90 min gradient. The column flow rate was maintained at 400 nL/min, with a column temperature of 40 °C. An electrospray voltage of 2 kV (versus the inlet of the mass spectrometer) was used.

Tandem mass spectra were processed by PEAKS Studio version X+ (Bioinformatics Solutions Inc., Waterloo, ON, Canada). PEAKS DB was set up to search the swissprot human databases (ver 201,907, 20,411 entries), assuming trypsin as the digestion enzyme. PEAKS DB were searched with a fragment ion mass tolerance of 0.02 Da and a parent ion tolerance of 7 ppm. Carbamidomethylation (C) and TMT 6plex (K, N-term) 229.16 were specified as the fixed modification. Oxidation (M) and Acetylation (Protein N-term) were specified as the variable modifications. Peptides were filtered by 1% FDR and 1 unique. Reporter ions were used to calculate the quantification ratio between samples. Normalization was calculated from the total intensity of all labels in all quantifiable peptides. Differently expressed proteins were filtered if their fold change were over 1.5 and contained at least 1 unique peptide with *p* < 0.05.

### 2.4. Bioinformation Analysis

Hierarchical cluster analysis was performed to find discrete groups with varying degrees of proteins in exosomes. This analysis was processed with the pheatmap package. Blast2GO version 4 was used for functional annotation of DEPs. A whole-protein sequence database was analyzed by BlastP using whole database and mapped and annotated with a gene ontology database. Statistically altered functions of different expressed proteins was calculated by Fisher’s exact test in BLAST2GO. The KEGG enrichment analysis of DEPs was processed by KOBAS (http://kobas.cbi.pku.edu.cn/ (accessed on 1 April 2020)). A volcano plot was used to plot significance versus fold-change on the y and x axes of differentially expression proteins (DEPs) (https://huygens.science.uva.nl/VolcaNoseR/ (1 December 2021)).

### 2.5. ELISA Analysis

Concentrations of Integrin-linked kinase 1 (ILK1) and Cluster of differentiation 14 (CD14) in plasma samples from 37 gastric cancer patients were determined by ELISA kits (CUSABIO, Wuhan, China), following the manufacture’s instruction. In brief, samples and standards were pipetted into the wells. After removing any unbound substances, a biotin-conjugated antibody specific for target protein was added to the wells. After washing, avidin-conjugated Horseradish Peroxidase (HRP) was added to the wells. Following a wash to remove any unbound avidin-enzyme reagent, a substrate solution was added to the wells and color developed in proportion to the amount of ILK-1 bound in the initial step. The color development was stopped, and the intensity of the color was measured.

### 2.6. Cell Lines and Transfection

Gastric cancer cell lines MKN45 and NCI-N87 were preserved in the Shanghai Institute of Digestive Surgery, Ruijin Hospital. Cells were cultured in RPMI-1640 medium with 10% fetal calf serum at 37 °C with 5% CO_2_. Lentiviral overexpression vectors of CD14 and ILK1 were purchased from Genomeditech (Shanghai, China). Stable transfected cells were selected and validated by quantitative reverse transcription-polymerase chain reaction (qRT-PCR), western blot with primary antibodies CD14 (1:1000, Ab-mart) and ILK1 (1:1000, Cell Signaling Technology, Danvers, MA, USA).

### 2.7. In Vitro Experiments and Western Blot

Gastric cells from the overexpression groups and control groups were collected, and single cell suspensions were prepared. The procedure of experiments was performed as previously described [13]. In brief, for cell cycle analysis, cells were fixed in 70% ice-cold ethanol and were stained with propidium iodide. Cells were subjected to fluorescence-activated cell sorting. For colony formation assay, 1000 cells were suspended in 4 mL full medium and were cultured in regular conditions to form single-cell colonies. After 2 weeks, colonies were fixed by 10% formalin and stained by crystal violet. The number and size of colonies were analyzed by ImageJ software (v1.53k, NIH). For migration assay, cells suspended in 100 μL serum-free medium were added into the upper chambers of Transwell (8 μm for 24-well plate, Millipore, Burlington, MA, USA), and those in the full medium were added into the lower chambers for 24 h. Cells were fixed by formalin and stained by 0.1% crystal violet. For western blot, primary antibodies E-cadherin (1:1000, ABclonal, Woburn, MA, USA), Vimentin (1:1000, Cell Signaling Technology) and Snail (1:1000, ABclonal) were incubated overnight at 4 °C. Secondary antibodies were incubated at room temperature. Protein bands were visualized by an infrared imaging system (LI-COR) and ECL substrate solution (NCM Biotech, Newport, RI, USA). GAPDH was used as loading control.

### 2.8. Single-Cell RNA Sequencing (scRNA-Seq) Analysis

Peripheral blood mononuclear cells (PBMCs) and cells in the ascites sample were isolated by density gradient centrifugation using Ficoll-Paque Plus medium (GE Healthcare, Chicago, IL, USA) and washed with Ca-/Mg-free PBS. Cells were resuspended by PBS to obtain a single-cell suspension. Single-cell suspensions were loaded onto microfluidic devices, and scRNA-seq libraries were constructed according to the Singleron GEXSCOPER protocol by GEXSCOPER Single-Cell RNA Library Kit (Singleron Biotechnologies, Köln, Germany) [14]. Pools were sequenced on Illumina HiSeq X with 150 bp paired-end reads. Raw reads were processed with fastQC and fastp to remove low-quality reads. Gene counts and UMI counts were acquired by featureCounts software (v2.0.1) [15]. Expression matrix files for subsequent analyses were generated based on gene counts and UMI counts. Genes expressed in more than 10% of the cells in a cluster and with average log (Fold Change) of greater than 0.25 were selected as DEGs by Seurat v3.1.2 FindMarkers, based on the Wilcox likelihood-ratio test with default parameters. The cell type identity of each cluster was determined from the expression of canonical markers found in the DEGs using the SynEcoSysR database. Heatmaps displaying the expression of markers used to identify each cell type were generated by Seurat v3.1.2 DoHeatmap. The InferCNV package was used to detect the CNVs in subclusters of epithelial malignant cells. Gene Ontology (GO) and Kyoto Encyclopedia of Genes and Genomes (KEGG) analyses were used with the “clusterProfiler” R package 3.16.1 [16]. Pathways with *p*_adj value less than 0.05 were considered significantly enriched.

### 2.9. Statistical Analysis

One-way ANOVA test (LSD post-hoc) was used to analyze quantitative data. The chi-square test was used to analyze the categorical variables. The cut-off value of exosome proteins was determined by a receiver operating characteristics (ROC) curve. *p* < 0.05 was considered statistically significant. Data analyses were performed using SPSS 22.0 software (Chicago, IL, USA).

## 3. Results

### 3.1. Clinical Characteristics of 12 AGC Patients with Organ-Specific Metastasis

The clinical characteristics of 12 chemo-naïve AGC patients are listed in Table 1. Patients were stratified into three groups according to their metastatic modes: L group (distant lymphatic metastasis), H group (hepatic metastasis) and P group (peritoneal metastasis). There were four patients in each group. There were 8 males and 4 females, with median age of 63 years old (ranging from 26 to 69 years old). The pathologic types of primary lesions of patients with peritoneal metastasis were all signet-ring cell carcinoma. Primary lesions of most patients were not resected at time of diagnosis.

### 3.2. Distinct Biological Functions of Differential Expression Proteins (DEPs) Detected in Plasma Exosomes from H Group and P Group

**Figure 1:**
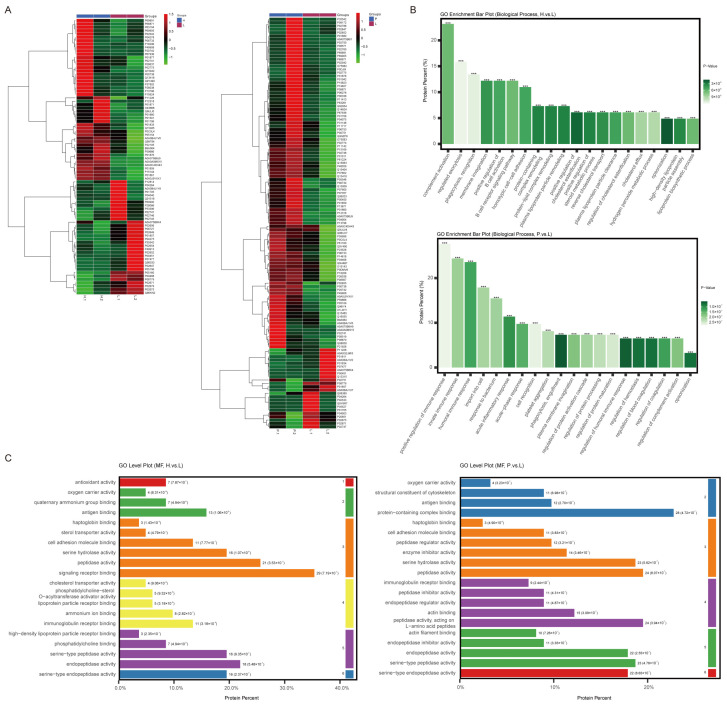
DEPs of plasma exosomes detected in AGC patients with different organ-specific metastasis and GO analysis of DEPs. (**A**): Heatmap of DEPs’ average values in H group vs. L group and P group vs. L group. (**B**): Biological processes of GO analysis enriched by DEPs (***: FDR ≤ 0.001). (**C**): Molecular functions of GO analysis enriched by DEPs.

Compared with exosome proteins detected in the L group, 48 high expression proteins (HEPs) and 34 low expression proteins (LEPs) were identified in the H group, while 99 HEPs and 24 LEPs were identified in the P group (Figure 1A, Table S2). The top-20 biological processes (BPs) of DEPs enriched by GO enrichment analysis in the H group and P group are illustrated in Figure 1B. For the H group, complement activation, regulated exocytosis, phagocytosis recognition, membrane invagination and positive regulation of B cell activation were the top-five enriched functions. For the P group, positive regulation of immune response, innate immune response, humoral immune response, import into cells and response to bacterium were the top-ranked enriched functions. Proteins involved in lipid and cholesterol metabolism, such as protein–lipid complex remodeling, plasma lipoprotein particle remodeling and positive regulation of cholesterol esterification, were mainly identified in the H group. Molecular function (MF) analysis showed that sterol transporter activity, cholesterol transporter activity and lipoprotein particle receptor binding were exclusively enriched in the H group, while structural constituents of the cytoskeleton, actin binding and actin filament binding were mainly enriched in the P group (Figure 1C).

### 3.3. Signaling Pathways Involved in Lipid Metabolism in the H Group and in Cytoskeleton Regulation in the P Group Were Identified

**Figure 2:**
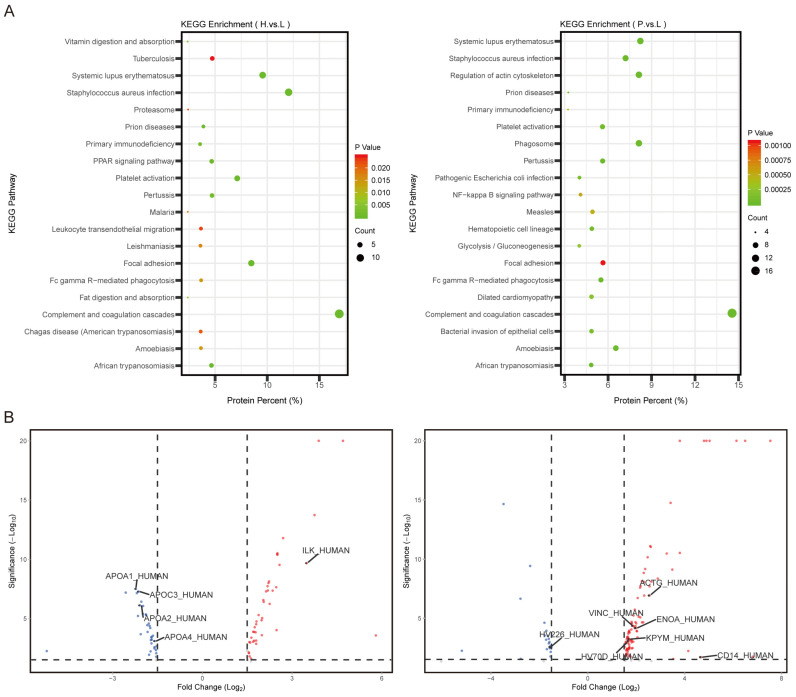
KEGG enrichment analysis of DEPs. (**A**): Signaling pathways of KEGG analysis enriched by DEPs. (**B**): Volcano plot of top-ranked DEPs from H group vs. L group and P group vs. L group.

DEPs from the H and P groups were further analyzed by KEGG enrichment analysis. In addition to common signaling pathways, DEPs from the H group could be enriched in those related to fat digestion and absorption, vitamin digestion and absorption and the PPAR signaling pathway. DEPs from the P group could be enriched in those related to the regulation of actin cytoskeleton, NF-κB signaling pathway and glycolysis/gluconeogenesis (Figure 2A). Top-ranked DEPs involved in these pathways from the two groups were illustrated using a volcano plot. ILK was identified as HEP in the H group, and APOA1, APOA2, APOC3 and APOA4 were identified as LEPs. For the P group, ACTG, VINC, ENOA, KPYM and CD14 were identified as HEPs, and HV226 was identified as LEP (Figure 2B).

### 3.4. Plasma ILK1 and CD14 Correlated with Hepatic and Peritoneal Metastasis in AGC, Respectively

**Figure 3:**
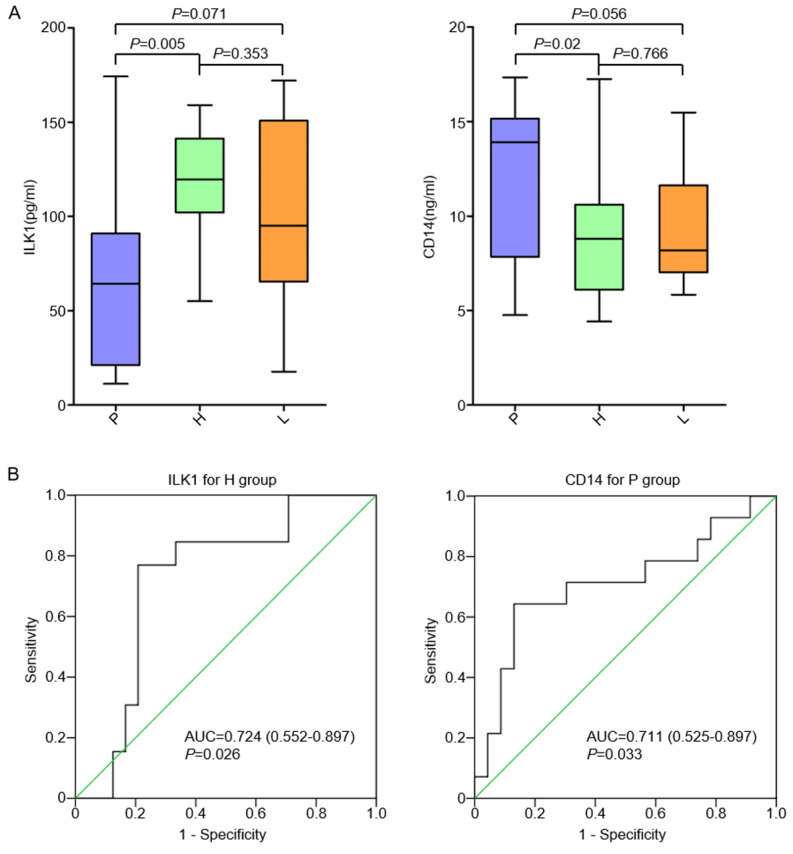
Correlation of plasma ILK1 and CD14 with organ-specific metastasis. (**A**): Plasma level of ILK1 and CD14 in AGC patients with different metastatic modes. (**B**): ROC curve to identify cut-off values of ILK1 and CD14.

Plasma samples were collected from 37 chemo-naïve AGC patients with organ-specific metastasis, including 10 patients with distant lymphatic metastasis (L group), 13 patients with hepatic metastasis (H group) and 14 patients with peritoneal metastasis (P group). The level of ILK1 in the H group was significantly higher than it was in the P group (*p* = 0.005), while it was similar in the L group (Figure 3A). The level of CD14 in the P group was significantly higher than it was in the H group (*p* = 0.02) and was also relatively higher than it was in the L group (*p* = 0.056), although the *p*-value was marginal (Figure 3A). Cut-off values of ILK1 and CD14 to identify patients with hepatic metastasis and peritoneal metastasis were determined by ROC curves. The cut-off value of ILK1 to indicate hepatic metastasis was 109.5 pg/mL, and AUC was 0.724 (95% CI 0.552–0.897, *p* = 0.026). The cut-off value of CD14 to indicate peritoneal metastasis was 11.6 ng/mL, and AUC was 0.711 (95% CI 0.552–0.897, *p* = 0.033) (Figure 3B). High ILK1 was found in 10 patients in the H group (10/13, 76.9%, *p* = 0.003), which was significantly higher than in the P group (2/14, 14.3%) or the L group (3/10, 30.0%). High CD14 was found in nine patients in the P group (9/14, 64.3%, *p* = 0.004), which was significantly higher than in the H group (1/13, 7.7%) or the L group (2/10, 20.0%) (Table 2).

### 3.5. Overexpression of CD14 and ILK1 Impacted Biological Behaviors in Gastric Cancer Cells

**Figure 4:**
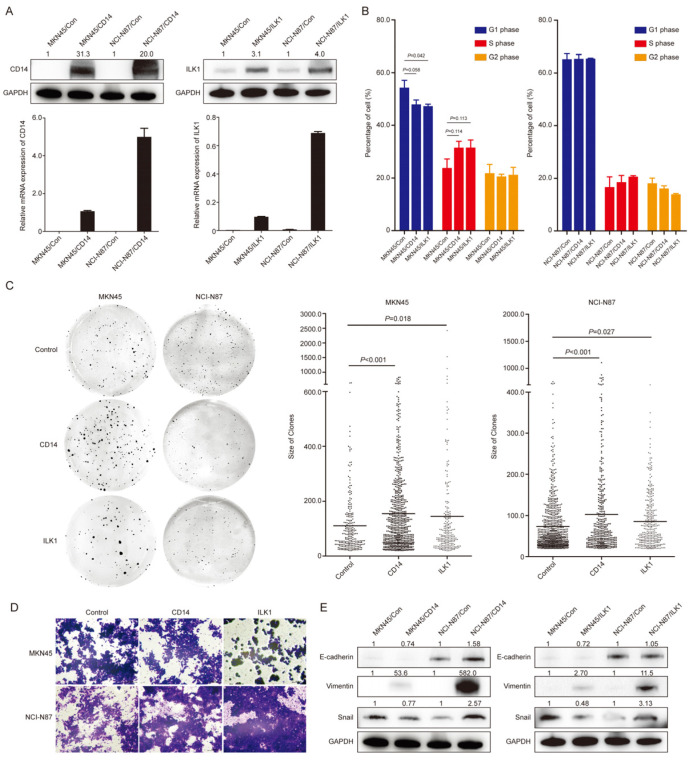
Overexpression of CD14 and ILK1 impacted biological behaviors in gastric cancer cells. (**A**): Expression of CD14 and ILK1 in MKN45 and NCI-N87 cells were detected by qRT-PCR and western blot. (**B**): Cell cycle analysis of CD14 and ILK1 overexpression cell models. (**C**): Representative images of colony formation assay. Colony size analysis was performed by ImageJ software. (**D**): Representative images of migration assay. (**E**): Expression of E-cadherin, Vimentin and Snail by western blot. Quantification data of western blot bands were analyzed by ImageJ software and compared with control groups. Original western blots have been presented in File S1.

CD14 and ILK1 were overexpressed in MKN45 and NCI-N87 cells, respectively, and were confirmed by qRT-PCR and western blot (Figure 4A). In cell cycle analysis, the proportions of cells in the G1 phase were decreased in MKN45/CD14 (*p* = 0.058) and MKN45/ILK1 (*p* = 0.042) cells (compared with MKN45/Con cells), while no significant differences were detected in NCI-N87 cells (Figure 4B). In colony formation assay, the number of colonies in MKN45/CD14 cells was higher than MKN45/Con (234.0 ± 41.2 vs. 122.5 ± 6.4, *p* = 0.007), while it was not different between MKN45/ILK1 and MKN45/Con (80.7 ± 12.9 vs. 122.5 ± 6.4, *p* = 0.156). The number of colonies in NCI-N87/CD14 (136.3 ± 52.9 vs. 262.7 ± 40.5, *p* = 0.007) and NCI-N87/ILK1 (118.3 ± 10.0 vs. 262.7 ± 40.5, *p* = 0.004) were both lower than NCI-N87/Con (Figure 4C). The average sizes of colonies were significantly increased in CD14 and ILK1 overexpression cells compared with control cells, both for MKN45 (154.6 ± 142.9 vs. 110.4 ± 108.8, *p* < 0.001; 144.6 ± 228.3 vs. 110.4 ± 108.8, *p* = 0.018) and NCI-N87 cells (102.7 ± 121.6 vs. 73.4 ± 64.9, *p* < 0.001; 85.4 ± 65.2 vs. 73.4 ± 64.9, *p* = 0.027, Figure 4C). For NCI-N87 cells, CD14 and ILK1 overexpression increased the numbers of cells penetrating the membrane of chambers, while this was not observed in MKN45 cells (Figure 4D). Expression of Vimentin protein was significantly higher in both CD14 and ILK1 overexpression cells. Increased expression of Snail was observed in NCI-87 cells, while it was not observed in MKN45 cells. E-cadherin levels were not changed (Figure 4E).

### 3.6. Immune Cells in Malignant Ascites in a Gastric Cancer Patient Showed High CD14 Expression

**Figure 5:**
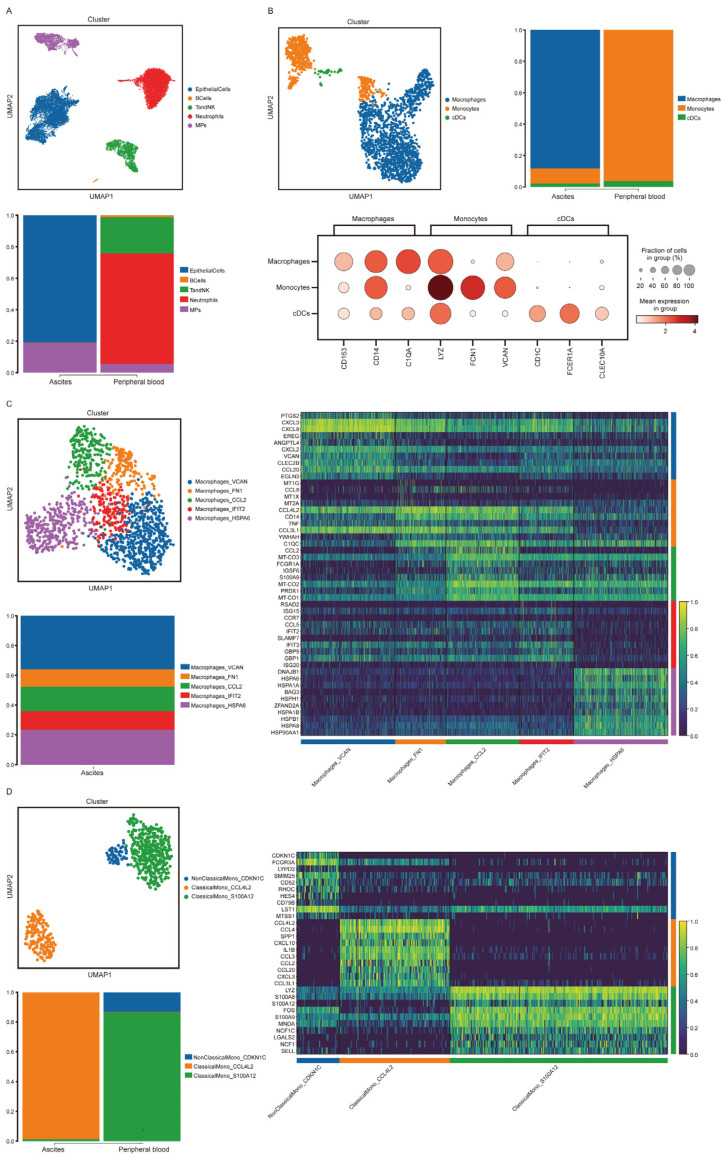
CD14 derived from subclusters of macrophages and monocytes in malignant ascites. (**A**): Cellular constitution of ascites sample and peripheral blood sample. (**B**): Cellular constitution of mononuclear phagocytes and cellular markers expression. (**C**): Subcluster analysis of macrophages in ascites samples. The heatmap illustrates the differential expression genes used to identify subcluster of macrophages. (**D**): Subcluster analysis of monocytes in ascites sample and peripheral blood sample. The heatmap illustrates the differential expression genes used to identify subcluster of monocytes.

A total of 8515 cells in the ascites sample and 8394 cells in the peripheral blood sample were identified. Epithelial cells, B cells, T and NK cells, neutrophils and mononuclear phagocytes (MPs) were annotated (Figure 5A). Malignant epithelial cells could be divided into eight cellular subclusters. No subcluster of malignant epithelial cells showed high expression of CD14 or ILK1 (Figure S3). Mononuclear phagocytes were divided into macrophages, monocytes and conventional dendritic cells, and no macrophages were found in the peripheral blood sample. High CD14 expression was shown in both macrophages and monocytes (Figure 5B). Macrophages in the ascites sample were subdivided into five subclusters and were annotated by the top-ranked expressed genes. High CD14 expression was detected in the subclusters Macrophage_FN1 and Macrophage_CCL2 (Figure 5C, Tables S3 and S4). Monocytes in both ascites and peripheral blood samples could be divided into three subclusters. High CD14 expression was detected in subcluster ClassicalMono_CCL4L2, which was the major part in ascites, compared with subcluster ClassicalMono_S100A12 and NonClassicalMono_CDKN1C in peripheral blood (Figure 5D, Tables S5 and S6).

### 3.7. High CD14 Expression in Macrophages and Monocytes Featured High Activation of Chemokine- and Cytokine-Mediated Signaling Pathways

**Figure 6:**
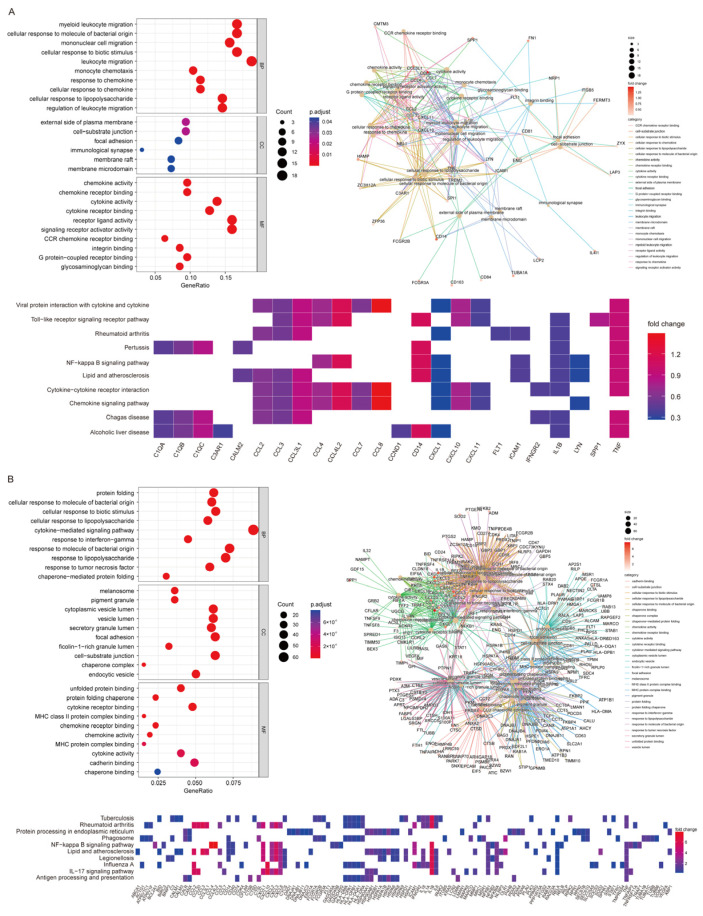
Biological functions analysis of high-expression DEGs of subclusters of macrophages and monocytes. (**A**): Dot plot and net plot of GO analysis of high-expression DEGs of Macrophage_FN1, and heat map of significantly enriched signaling pathways. (**B**): Dot plot and net plot of GO analysis of high-expression DEGs of ClassicalMono_CCL4L2, and heat map of significantly enriched signaling pathways.

Biological functions of immune cell subclusters with high CD14 expression, including Macrophage_FN1 and ClassicalMono_CCL4L2, were further analyzed based on their high-expression DEGs. For Macrophage_FN1, GO enrichment analysis showed that monocyte chemotaxis and cellular response to chemokine were ranked in the top-10 biological processes (BPs). Chemokine activity and cytokine activity were its top-ranked molecular functions (MFs) (Figure 6A). For ClassicalMono_CCL4L2, the cytokine-mediated signaling pathway was also ranked among the top-10 BPs, as well as chemokine activity and cytokine activity in MFs (Figure 6B). KEGG enrichment analysis showed that chemokines including CCL2, CCL3, CCL3L1, CCL4, CXCL1 and CXCL10 were both detected as high-expression DEGs in Macrophage_FN1 and ClassicalMono_CCL4L2, and played important roles in multiple biological functions and signaling pathways (Figure 6A,B).

## 4. Discussion

In the present study, we showed that the biological functions of plasma exosome proteins from AGC patients with different organ-specific metastases were distinct. Exosome proteins involved in lipid metabolism were identified in patients with hepatic metastasis, while proteins involved in actin regulation and glycolysis/gluconeogenesis were identified in patients with peritoneal metastasis. Plasma levels of ILK1 and CD14 were correlated with hepatic and peritoneal metastasis, respectively. Overexpression of CD14 and ILK1 could increase colony formation ability and expression of epithelial–mesenchymal transition (EMT) markers in gastric cancer cells. By using scRNA-seq, CD14 was found to be derived from subclusters of macrophages and monocytes in ascites which featured high activation of chemokine- and cytokine-mediated signaling pathways.

The exosome is an important class of extracellular vehicles (EVs), measuring 30–100 nm in diameter. During cancer progression and metastasis, bioactive substances packed in exosomes, including DNAs, microRNAs, long non-coding RNAs (lncRNAs) and proteins can be transmitted among tumor cells, surrounding the tumor microenvironment and distant organs to regulate critical biological functions [17]. The exosome also participates in the process of organ-specific metastasis in cancer cells. Hoshino et al. found that distinct integrin expression patterns in tumor-derived exosomes from organ-tropic tumor cells associated with metastatic organotropism [18]. Meanwhile, with the features of high stability and huge amount in body fluid of patients, exosomes also have the potential to be biomarkers for cancer diagnosis and surveillance based on liquid biopsy [19]. Biological functions of exosome cargos have been investigated in gastric cancer, while the roles of exosome proteins in regulating organ-specific metastasis have not been fully analyzed [11,20]. In the present study, by using GO and KEGG enrichment analysis, biological functions of exosome differential proteins in AGC patients with hepatic- or peritoneal-specific metastasis were found to be distinct from those in patients with distant lymphatic metastasis, which showed different molecular mechanisms utilized by cancer cells to metastasize to target organs.

Abnormal metabolic reprogramming is one of the hallmarks of cancer [21]. Different metabolic pathway alterations were found in the present study in patients with hepatic and peritoneal metastasis. Exosome proteins involved in lipid metabolism were found to be correlated with hepatic metastasis, while glycolysis/gluconeogenesis was identified in patients with peritoneal metastasis. Correlations between fatty acid metabolism and lymphatic metastasis in cancer cells have been demonstrated [22]. Up-regulation of fatty acid oxidation was found in gastric cancer cells with high capability of lymphatic metastasis and this could be transmitted by exosomes [23]. A relationship between abnormal lipid metabolism and hepatic metastasis was also found in breast cancer and melanoma. Pathological accumulation of lipid in the liver could significantly increase hepatic metastasis [24]. Cholesterol synthesis was significantly correlated with metastasis of hepatocellular carcinoma by attenuating CD44-Ezrin binding, which could inhibit cell migration [25]. Low expression of apolipoproteins including APOA1, APOA2, APOA4 and APOC3 in plasma exosomes of patients with hepatic-specific metastasis indicated an impaired function of lipid metabolism, which could participate in hepatic metastasis in gastric cancer cells.

Glycolysis/gluconeogenesis was exclusively enriched by DEPs in patients with peritoneal-specific metastasis. The Warburg effect—which enhances glycolysis under aerobic conditions—is closely related to the invasion and metastasis of gastric cancer [26]. During the development of peritoneal metastasis, tumor cells shed into the peritoneal cavity face the stress of hypoxia and anoikis. Metabolic reprogramming, mediated by glycolytic enzymes and hypoxia inducible factor-1α, is critical for tumor cells to survive and form peritoneal metastases [27]. In the present study, alpha-enolase (ENO1) was identified as the HEP of patients in the P group, which is a key regulatory enzyme of glycolysis. Emerging evidence demonstrates that ENO1 is also directly involved in tumor cell progression and metastasis [28]. Furthermore, regulation of the actin cytoskeleton, which was one of the features of epithelial mesenchymal transition (EMT), was also mainly enriched by DEPs identified in plasma exosomes from patients with peritoneal metastasis. The critical role of EMT during the processes of peritoneal metastasis in gastric cancer cells has been demonstrated [7,29]. Therefore, according to our results, metabolic reprogramming, depending on different nutrients as well as specific biological functions, could drive organ-specific metastasis in gastric cancer cells.

Two DEPs, ILK1 and CD14, were found to be correlated with hepatic and peritoneal metastasis in the present study, respectively. Currently, the roles of ILK1 and CD14 in the plasma exosomes of gastric cancer patients have not been fully reported. ILK1 is a kind of serin/threonine kinase which functions as an adaptor and mediator protein, linking the extracellular matrix with downstream signaling pathways, including RAS/RAF/ERK, PKB/Akt and NF-κB [30]. ILK was overexpressed in many cancers and could regulate cell migration, metastasis and proliferation of cancer cells. In gastric cancer cells, ILK knockdown could inhibit cell growth and invasion by impairing the ERK1/2/NF-κB pathway activation and F-actin assembly [31]. Overexpression of ILK1 in tumor cells enhanced anchorage-independent survival, which was critical for hematogenous metastasis [32]. Cluster of differentiation 14 (CD14) protein, which was correlated with peritoneal metastasis in gastric cancer cells in the present study, was a coreceptor of bacterial lipopolysaccharide-mediating innate immune response and was associated with Helicobacter pylori infection and gastric carcinogenesis [33]. Li et al. reported that CD14 knockdown could inhibit EMT and invasive capacity of gastric cancer cells. CD14 could act via MyD88, TIRAP and TRAF6, also leading to NF-κB activation [34]. In the present study, by establishing CD14- and ILK1-overexpressing gastric cancer cell models, the size of colonies was significantly increased more than in control cells, which suggested CD14 and ILK1 could promote outgrowth of metastasis in gastric cancer. Expression of Vimentin and Snail, the makers of EMT, were increased in CD14- and ILK1-overexpressing cells, as well as the migration ability of NCI-N87 cells. Although changes of phenotypes were not fully identical in MKN45 and NCI-N87 cells, our results showed that CD14 and ILK1 could participate in regulating critical biological behaviors of cancer cells during metastasis which were outgrowth and EMT.

Exosomes can be secreted by nearly all cell types. Therefore, to clarify the origins of plasma exosome proteins, a peripheral blood sample and an ascites sample from one patient with peritoneal metastasis were intensively analyzed by scRNA-seq. CD14 expression was mainly identified in immune cells rather than cancer cells. However, as a surface antigen of macrophages and monocytes [35], its expression in immune cells was heterogenous. High expression of CD14 was detected in several subclusters of macrophages and monocytes in the ascites samples, while the major subcluster of monocytes in peripheral blood samples showed relatively low CD14 expression. Further biological function analysis showed that high-expression genes in those high-CD14 immune cells were enriched in signaling pathways of chemokine- and cytokine-mediated activities. Chemokines could directly induce tumor cells metastasis, which had been recognized decades ago. Immune cells such as M2 macrophages in the tumor microenvironment were the major source of chemokines as well as cytokines [36]. Those results suggested that enrichment of those immune cells in malignant ascites of gastric cancer patients could promote the peritoneal metastasis.

There were several limitations in our study. The sample size of patients was relatively small in our cohort. In clinical practice, sample collection from patients with organ-specific metastasis is relatively difficult, since patients with multi-organ metastasis are more common. Therefore, to validate DEPs detected in the primary cohort, samples from 37 patients were collected as a validation cohort. Due to the difficulty in identifying the cellular origin of plasma exosomes, we used scRNA-seq to analyze the molecular characteristics of both tumor cells and stromal cells in a malignant ascites and a peripheral blood sample, which could help us to identify the potential origin of plasma exosome proteins. In our future study, the biological functions of ILK1 and CD14 in driving organ-specific metastasis in gastric cancer cells will be further investigated.

## 5. Conclusions

In summary, biological functions of plasma exosome proteins among AGC patients with different metastatic modes were distinct. ILK1 and CD14 were correlated with organ-specific metastasis and participated in regulating biological behavior sin gastric cancer cells. The plasma exosome CD14 was mainly derived from immune cells in malignant ascites, which could promote peritoneal metastasis through chemokine- and cytokine-mediated signaling pathway.

## Figures and Tables

**Table 1:** Clinical information of 12 AGC patients.

Group	Case	Gender	Age	Site	Pathology	Primary Lesion Resection
Lymph node metastasis	L-1	M	65	Corpus	Adenocarcinoma	Yes
	L-2	M	69	Corpus	Adenocarcinoma	No
	L-3	M	65	Corpus	Adenocarcinoma	No
	L-4	F	69	Corpus	Adenocarcinoma	No
Hepatic metastasis	H-1	M	52	Cardiac	Adenocarcinoma	No
	H-2	M	66	Corpus	Poorly differentiated carcinoma	No
	H-3	M	62	Antrum	Poorly differentiated carcinoma	No
	H-4	M	66	Antrum	Adenocarcinoma	No
Peritoneal metastasis	P-1	M	54	Corpus	Poorly differentiated carcinoma, partial signet-ring cell carcinoma	No
	P-2	F	44	Cardiac	Poorly differentiated carcinoma, partial signet-ring cell carcinoma	Yes
	P-3	F	57	Corpus	Adenocarcinoma, partial signet-ring cell carcinoma	No
	P-4	F	26	Corpus	Signet-ring cell carcinoma	No

**Table 2:** Correlation between exosome proteins and organ-specific metastasis in AGC.

	ILK1 n (%)			CD14 n (%)		
Group	Low	High	*p* Value	Low	High	*p* Value
P	12 (85.7)	2 (14.3)	0.003	5 (35.7)	9 (64.3)	0.004
H	3 (23.1)	10 (76.9)		12 (92.3)	1 (7.7)	
L	7 (70.0)	3 (30.0)		8 (80.0)	2 (20.0)	

## Data Availability

Data can be accessed upon request to the corresponding author: Jun Zhang (junzhang10977@sjtu.edu.cn).

## Supplementary Materials

The following supporting information can be downloaded at: https://www.mdpi.com/article/10.3390/cancers15153986/s1, Figure S1: Representative CT images of patients with distant lymphatic, hepatic and peritoneal metastasis; Figure S2: Expression of exosome markers and particle size detection by electron microscopy and nanoparticle tracking analysis; Figure S3: Cellular subclusters analysis of malignant epithelial cells in ascites. A. Cellular markers expression of subclusters of malignant epithelial cells. B. Differential expression genes of subclusters of malignant epithelial cells; Table S1: Diameter and concentration of exosomes in samples; Table S2: Differentially expressed proteins; Table S3: Differential expression genes of subcluster Macrophages_FN1; Table S4: Differential expression genes of subcluster Macrophages_CCL2; Table S5: Differential expression genes of subcluster ClassicalMono_S100A12; Table S6: Differential expression genes of subcluster NonClassicalMono_CDKN1C. File S1: Original western blots.
