# Supplementary material for: Plasma Exosome Proteins ILK1 and CD14 Correlated with Organ-Specific Metastasis in Advanced Gastric Cancer Patients
Source: Cancers (Basel). 2023 Aug 5;15(15):3986. doi: 10.3390/cancers15153986 (PMC10417498; doi:10.3390/cancers15153986)
Supplement: Supplementary file 1 [file cancers-15-03986-s001.zip › Supplementary Table S1.pdf]

Supplementary Table 1 Diameter and concentration of exosomes in samples

Sample	Diameter (nm)	Concentration (/ml)
H1_2	93	3.20×10^{10}
P1_3	97.4	2.90×10^{11}
L1_3	86.8	1.60×10^{11}
L2_1	99.7	3.30×10^{11}
