# Supplementary figures and images for: Plasma Exosome Proteins ILK1 and CD14 Correlated with Organ-Specific Metastasis in Advanced Gastric Cancer Patients
Source: Cancers (Basel). 2023 Aug 5;15(15):3986. doi: 10.3390/cancers15153986 (PMC10417498; doi:10.3390/cancers15153986)

L-4

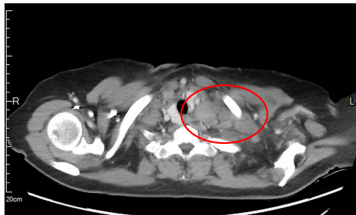

H-1

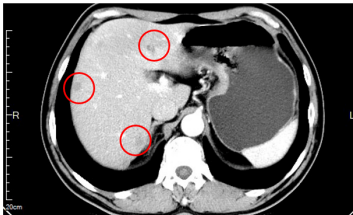

P-4

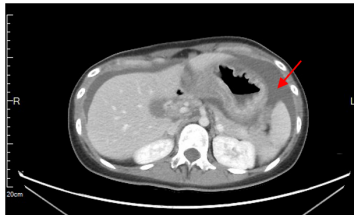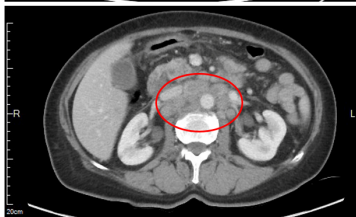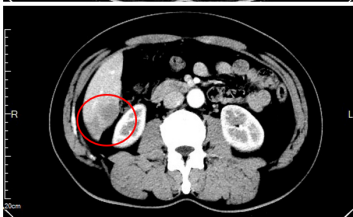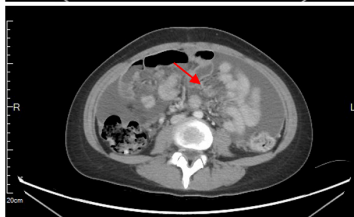

Supplement: Supplementary file 1 [file cancers-15-03986-s001.zip › Figure S1.pdf]

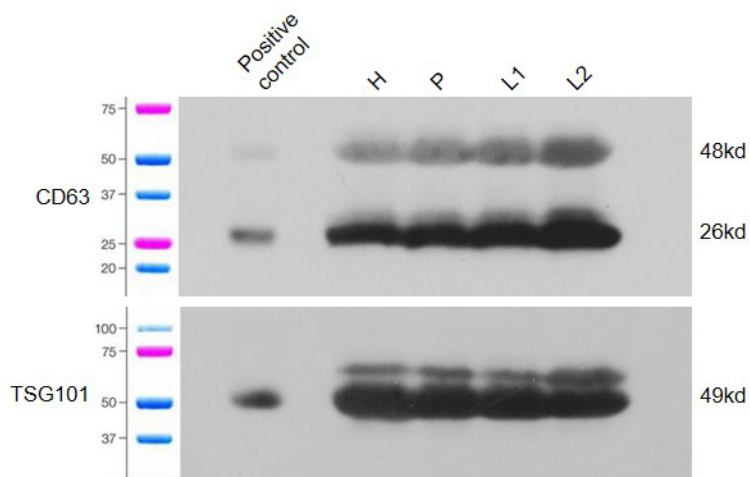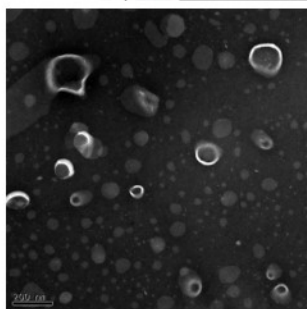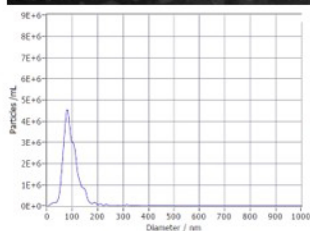

L

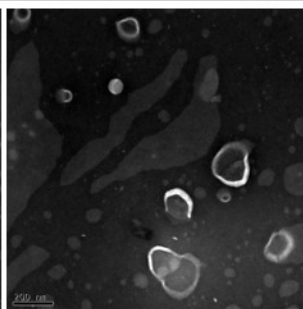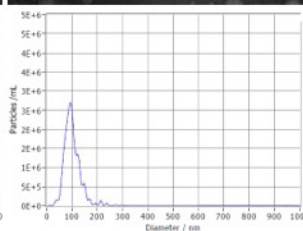

H

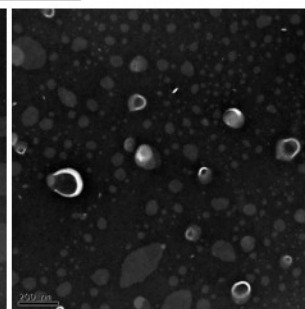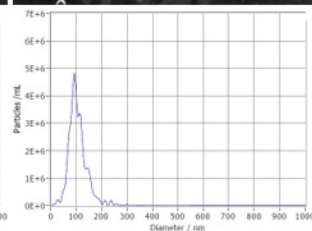

P

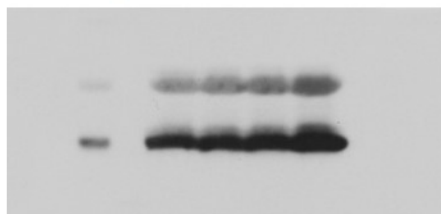

CD63

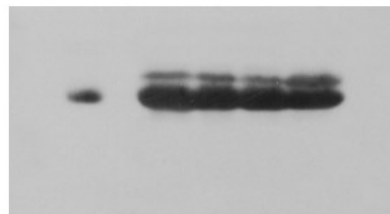

TSG101

Supplement: Supplementary file 1 [file cancers-15-03986-s001.zip › Figure S2.pdf]

A

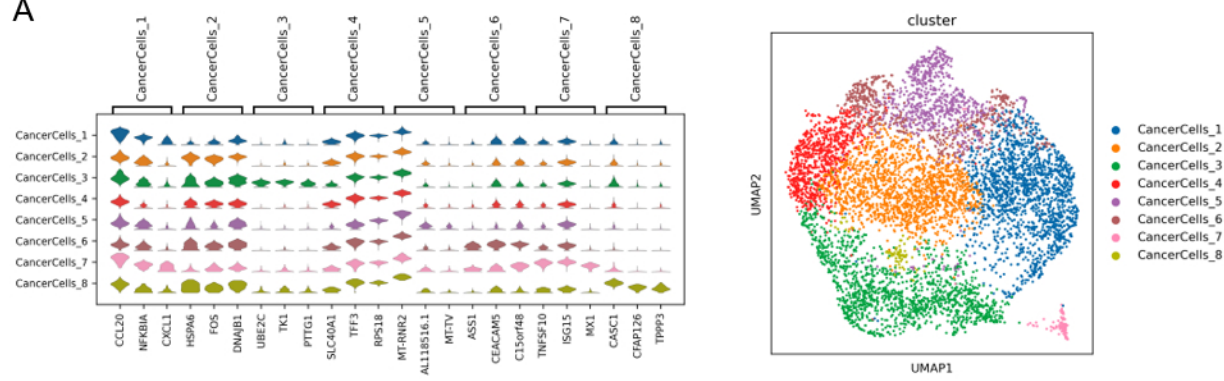

B

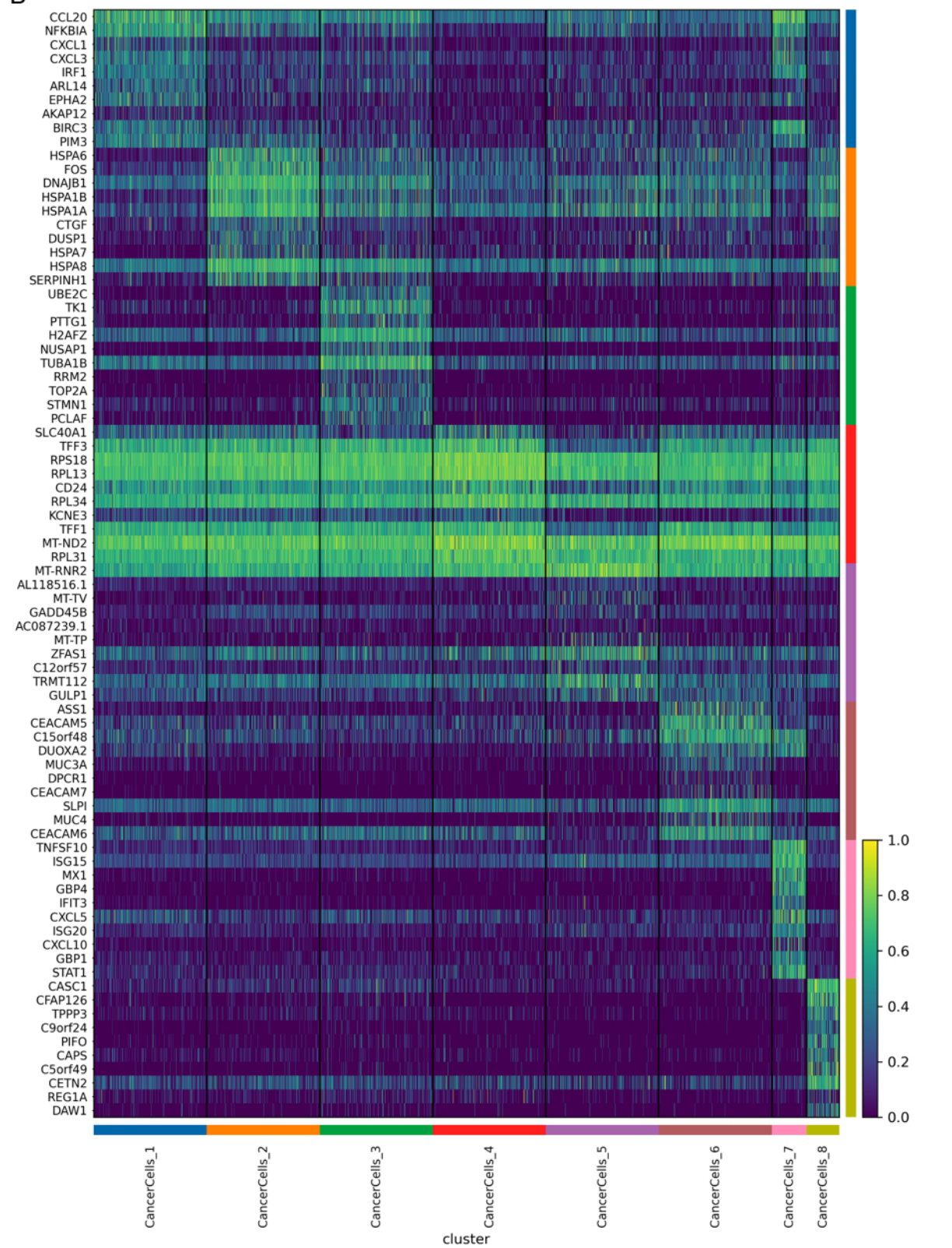

Supplement: Supplementary file 1 [file cancers-15-03986-s001.zip › Figure S3.pdf]

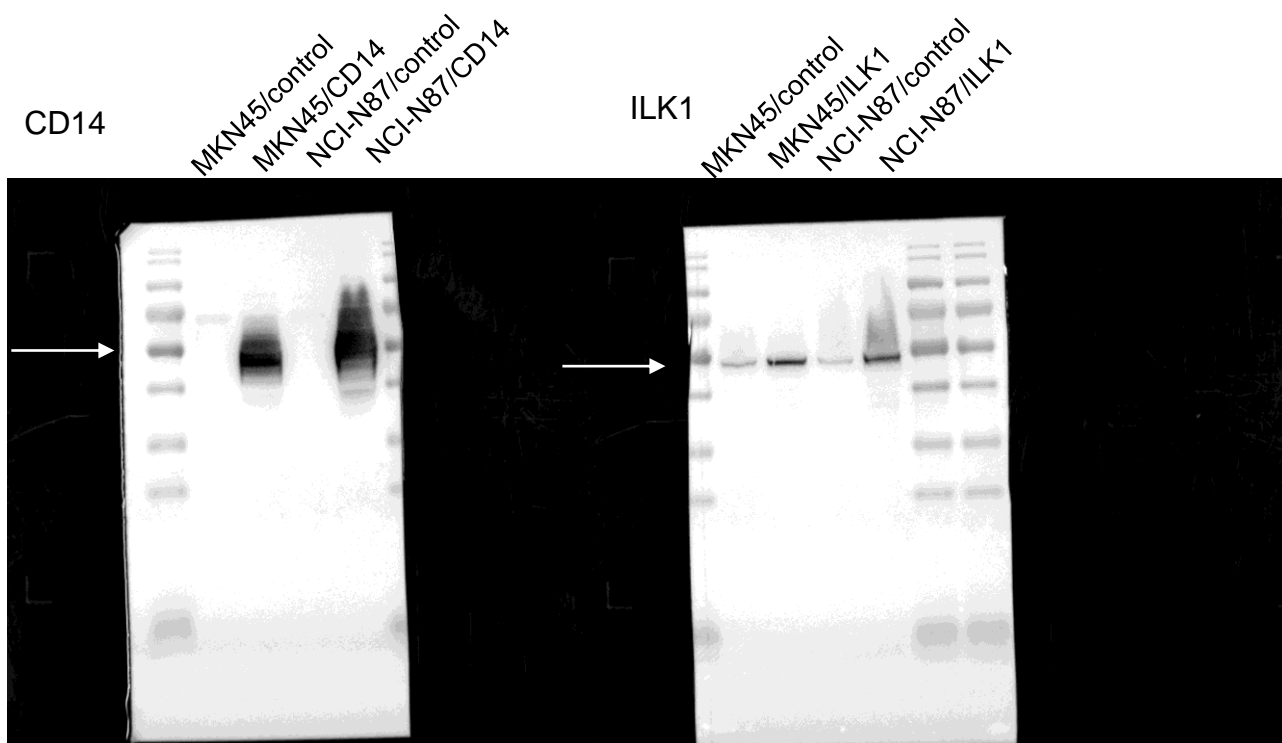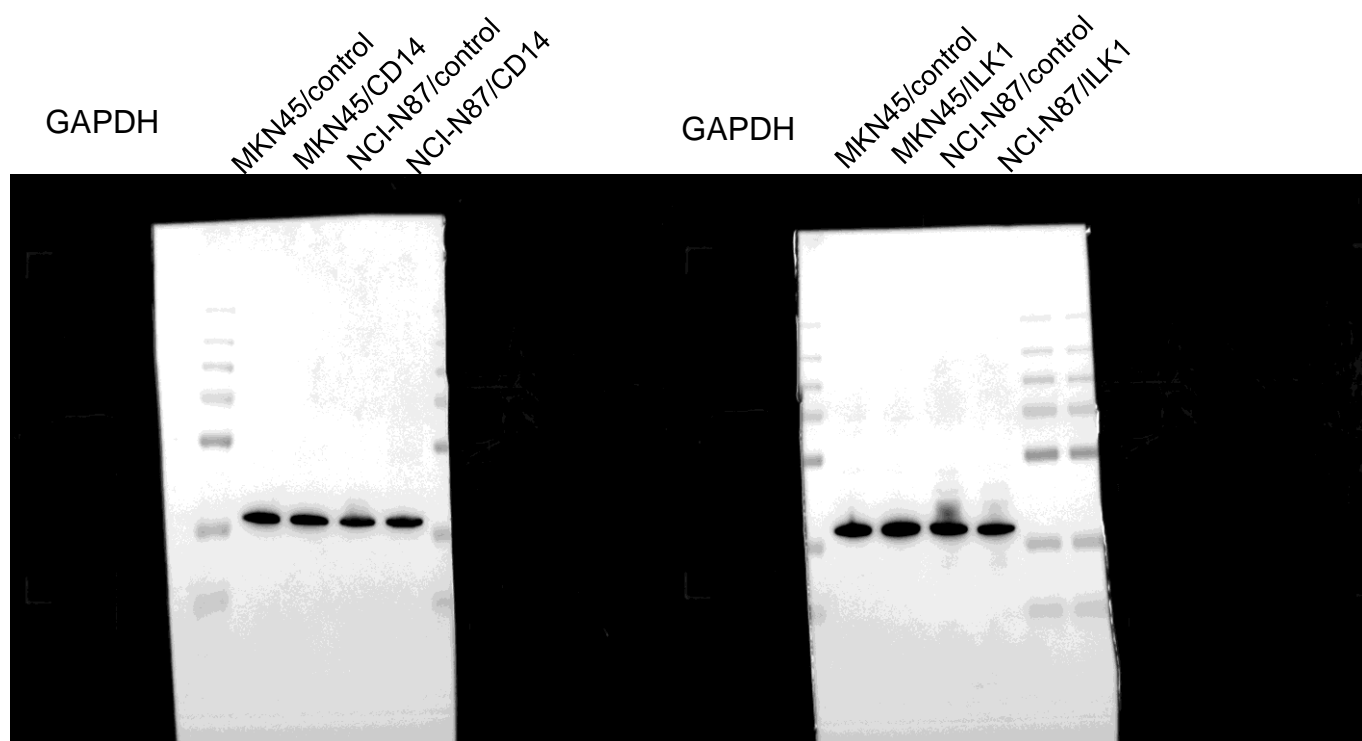

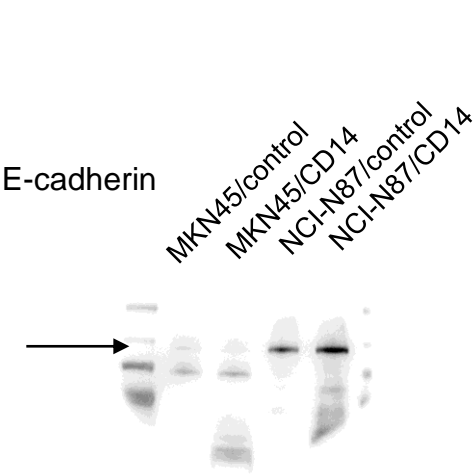

Figure 4E

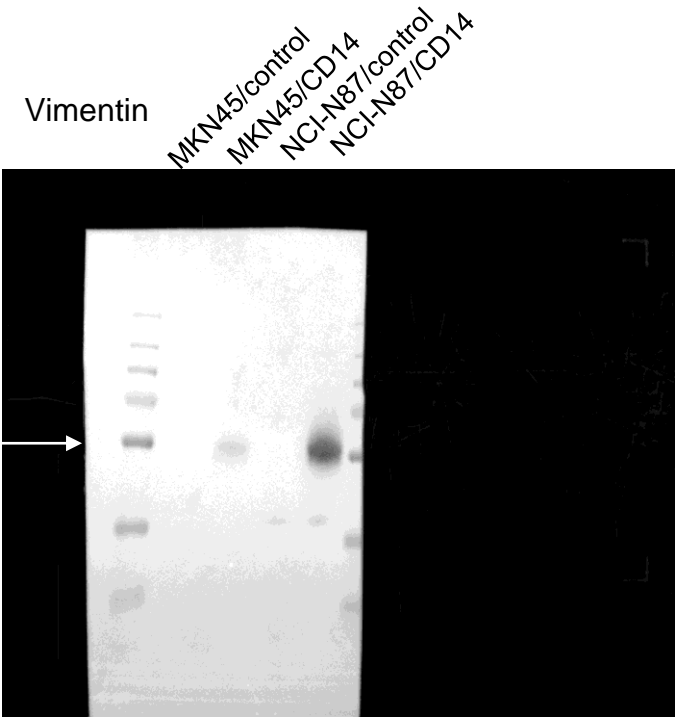

Figure 4E

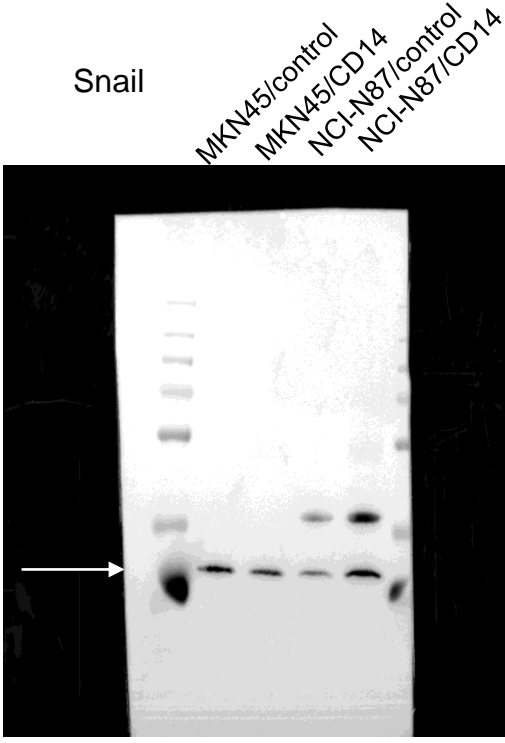

Figure 4E

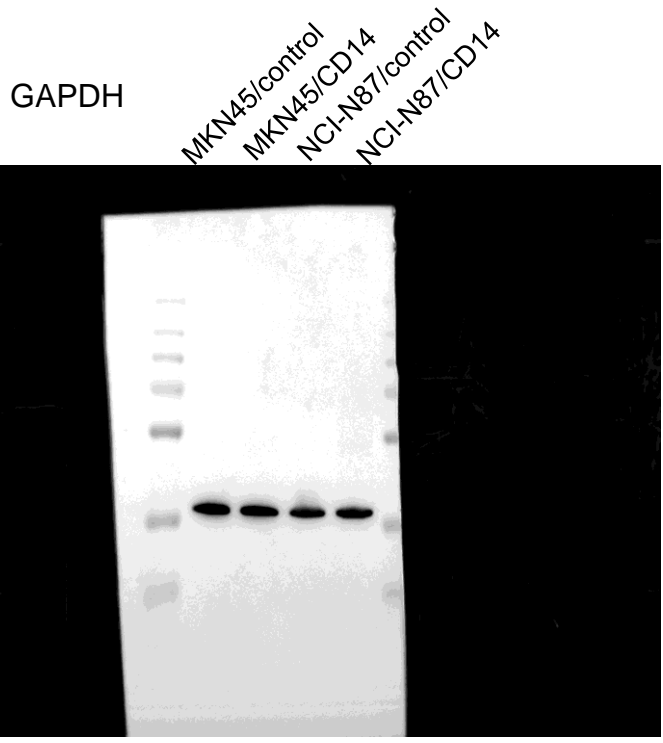

Figure 4E

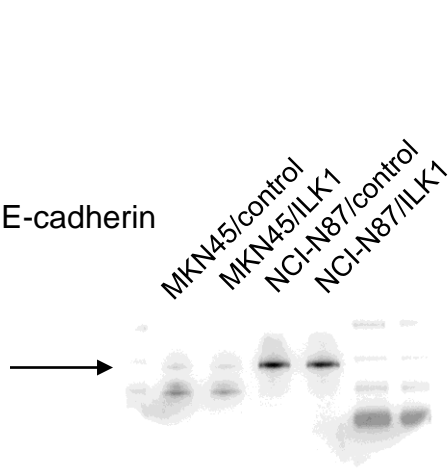

Figure 4E

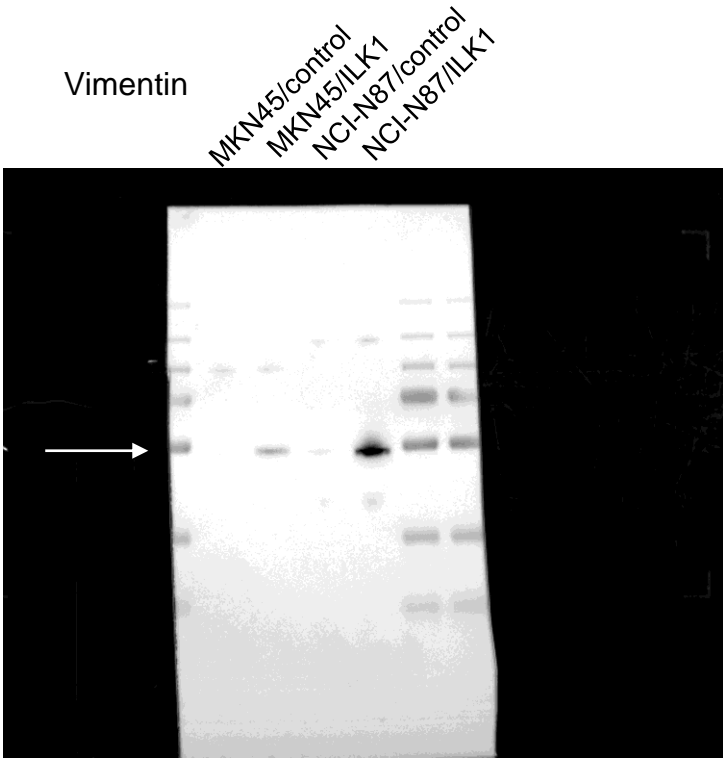

Figure 4E

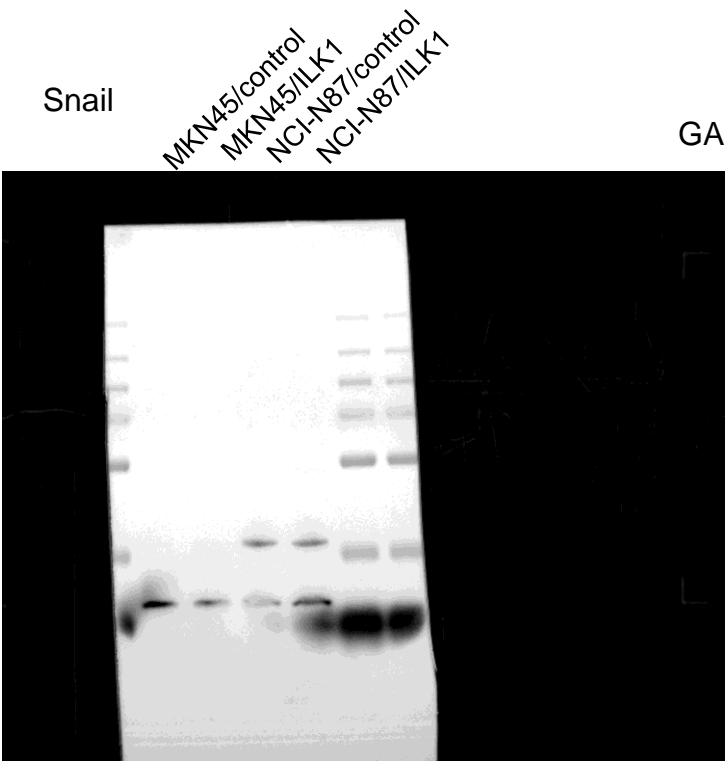

Figure 4E

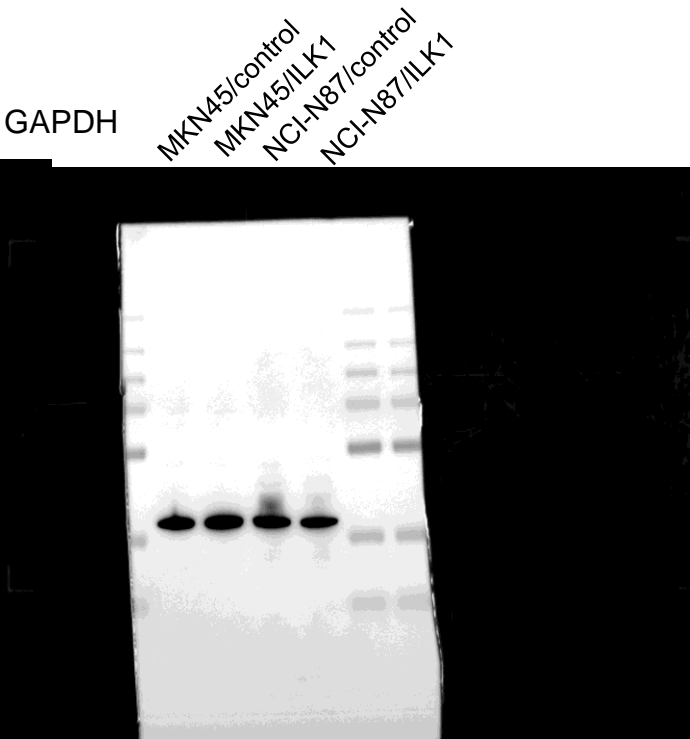

Figure 4E

Supplement: Supplementary file 1 [file cancers-15-03986-s001.zip › File S1. Original Images for Gels.pdf]
